# Recovery Characteristics in Neonates Following General Anesthesia: A Retrospective Chart Review

**DOI:** 10.7759/cureus.16126

**Published:** 2021-07-02

**Authors:** David Fanelli, Daniel Kim, Tonya S King, Gregory E Weller, Priti G Dalal

**Affiliations:** 1 Anesthesiology and Perioperative Medicine, Penn State Health Milton S. Hershey Medical Center, Hershey, USA; 2 Epidemiology and Public Health, Penn State College of Medicine, Hershey, USA

**Keywords:** postoperative apnea, neonatal, post operative monitoring, general anesthesia

## Abstract

Introduction

Preterm babies increasingly survive the neonatal period as a result of advanced care practices. Accordingly, anesthesiologists are likely to encounter these patients with greater frequency. Ex-premature infants and term neonates are known to have an increased risk of post-operative apneas following surgery and anaesthesia.

Methods

Following approval from the Institutional Review Board, we performed a retrospective chart review of neonates 0-28 days of age who underwent general anaesthesia procedures over two years. Data collected included age days, sex, weight, gestational age, American Society of Anaesthesiologists (ASA) physical status, type of anaesthetic (general/regional/spinal), airway management, surgical procedure, intraoperative adverse events, duration of anaesthesia, medications administered, post-operative recovery location, the occurrence of apneic events, medical co-morbidities, duration of post anaesthesia care unit (PACU) admission, a requirement for neonatal intensive care unit (NICU) admission, and duration of hospital admission.

Results

A total of 239 charts were reviewed from January 1, 2015, to December 31, 2016. Ninety-five cases were excluded for required postoperative mechanical ventilation. For the remaining 144 cases, the mean age was 12.8 days, 65% male, 35% female, mean gestational age 38.6 weeks, mean post-menstrual age 40.5 weeks, mean ASA status 3.5, and mean weight 3.46 kg. Post-operative apnea was observed in two neonates (1.4%).

Risk factors for postoperative apnea included lower gestational age at birth (median 37.5 vs. 39.1 weeks, p=0.26), lower post-menstrual age (median 38.5 vs. 41.0 weeks, p=0.18), and lower weight (median 2.8 vs. 3.5kg, p=0.27), respectively. ASA classification, preoperative anaemia, and known pathology were all significant risk factors for apnea (p<0.05). Significant factors from the bivariate analysis were preoperative anaemia, known pathology, age, duration of anaesthesia, weight, intraoperative fentanyl, and amount of neuromuscular blocker. Age and preoperative anaemia were significant predictors for recovery location. The odds of going to PACU vs NICU/PICU for post-operative recovery were 7.4 times greater for every two weeks greater age (95% CI=(2.80-19.31), p<0.001).

Conclusion

This study corroborates previous findings of predictive risk factors for post-anaesthesia apnea in preterm and term neonates. Previously reported risk factors, including low gestational/post-menstrual age, lower weight, and intraoperative narcotic use, were likely contributors to one of the apneic events in our study. A larger sample size is warranted to confirm a valid predictive model. Standardized universal guidelines would be useful in eliminating local variation in PACU monitoring and discharge criteria in this vulnerable age group.

## Introduction

The prevalence of infants born preterm is estimated at 8-10% worldwide [1]. With improvements in neonatal care, the number of these infants that survive continues to increase [1,2]. Ex-premature infants and term neonates have an increased risk of post-operative apneas following surgery and anaesthesia [3-7]. Several factors contribute to the increased susceptibility to apnea in infants, including immaturity of central respiratory mechanisms and musculature and a relatively higher basal metabolic rate [3,8]. Furthermore, infants are sensitive to the effects of anaesthetics and sedatives. In combination, these factors lead to an overall higher risk of apnea and bradycardia in these patients [3,9]. Amongst the most significant sequela of these cardio-respiratory insults are negative neurodevelopmental outcomes [10]. For example, literature assessing cerebral hemodynamics during a single apneic episode in infants has demonstrated decreased cerebral blood volume and cerebral blood flow velocity [1]. Accordingly, quantifying and decreasing the incidence of these episodes is of critical importance.

Current literature suggests the risk period for post-operative apneas and bradycardias may be up to 60 weeks post-menstrual age in premature infants and up to 45 weeks in term infants [11,12]. The previously described potential risk factors include post-menstrual age, weight, anaemia, administration of general anaesthesia, opioid administration intraoperatively, and procedure duration [12,13]. However, decisions regarding post-operative monitoring and discharge for patients with these risk factors are determined at the institutional level. Each hospital develops its criteria for monitoring and discharges from the hospital [14]. We employ a standardized protocol for post-operative management and monitoring for post-anaesthesia apneas in infants who undergo outpatient procedures (Appendix 1). Based on our protocol, all infants who are ex-premature and less than 60 weeks post-menstrual age and term infants less than 45 weeks post-menstrual age are admitted for apnea/bradycardia monitoring post-operatively minimum of 12 hours. For outpatient procedures, a 12-hour event-free period is required before discharge to home. Similar monitoring criteria are used in inpatient ex-premature infants < 60 weeks post-menstrual, and term infants < 45 weeks undergoing anaesthesia are also monitored for apnea/bradycardia post-operatively for at least 12-hours. The primary aim of this study was to audit the recovery characteristics in neonates after general anaesthesia at our Children’s Hospital. Our secondary aim was to identify the risk factors for the observed outcomes and adverse events.

## Materials and methods

Following Institutional Review Board approval with designation as non-human research, we performed a retrospective chart review of preterm and term neonates, 0-28 days of age who underwent general anaesthesia procedures, over two years from January 1, 2015, to December 31, 2016. Our Electronic Medical Record (EMR, Cerner Copyright © 2021 Cerner Corporation, MO, USA) was used to identify and retrieve the data using age-based search criteria for neonates (i.e. age <28 days). Patients were excluded if they were already tracheal intubated at the anaesthetic time or remained intubated following the anaesthetic. The data collected included age in days, sex, weight, gestational age, American Society of Anesthesiologist (ASA) physical status, type of anaesthetic (general/regional/spinal), airway management, surgical procedure, intraoperative adverse events, duration of anaesthesia, medications administered, post-operative recovery location, the occurrence of apneic/bradycardic events, history of medical co-morbidities, duration of Post Anesthesia Care Unit (PACU) admission, required neonatal intensive care unit (NICU) admission, and duration of hospital admission. 

Data regarding respiratory and cardiovascular complications were noted. Data included bradycardia (heart rate <80/min), oxygen desaturation (<90%), and apnea (visual observation of cessation of breathing documented in nursing notes or the EMR). The REDCap (Vanderbilt, USA) electronic data management tool was used to store and manage the data [15,16].

Statistical Analysis

Results are reported in the median, first, and third quartiles, and Wilcoxon p-values. The differences in characteristics between those with and without apnea were evaluated utilizing Wilcoxon rank-sum tests for continuous variables. To identify significant predictors of the secondary outcome recovery location, i.e. NICU vs PACU, bivariate analysis using chi-square tests for categorical variables and logistic regression for continuous variables was performed to evaluate each potential predictor separately. In addition, a multivariable logistic regression model was used to evaluate the factors predictive of recovery location in the bivariate analysis. A manual backward selection procedure was used to eliminate predictors which were no longer significant in the presence of the other factors until all predictors in the model were significant with p<0.05. Results are reported in unadjusted and adjusted odds ratios, 95% confidence intervals, and p-values. Statistical analysis was performed using SAS version 9.4 (SAS Institute, Cary NC), and p<0.05 was considered statistically significant.

## Results

Two hundred thirty-nine charts were reviewed from January 1, 2015, to December 31, 2016. Of these, 95 cases met exclusion criteria. Of the remaining 144 cases, the mean age was 12.8 days [±9.5, range 0-28 days], 65% male, 35% female, mean gestational age 38.6 weeks [± 1.8, range 32.3-40.6 weeks], mean post-menstrual age 40.5 weeks [±2.6, range 32.5-51.5 weeks], median ASA status was 2 [IQR 1.75-3, 1.25] and mean weight 3.46 kg [±0.77, range 1.91-7.01 kg]. A total of 20 out of 144 [13.8%] neonates were preterm (gestational age <37 weeks). Post-operative apnea was documented in two out of 144 neonates (1.4%).

The first term neonate was ASA physical status 1, 40-week post-menstrual age male on day of life seven who presented for placement of central venous catheter placement to administer anti-viral therapy for Herpes simplex infection. The nurse noted that the neonate sustained multiple apneic episodes (total 7). The shortest episode of apnea was seconds, while the longest episode lasted approximately 30 seconds with a nadir pulse oximetry value of <60%. In each instance, saturations improved following stimulation and administration of supplemental nasal oxygen. There were no associated bradycardia episodes documented with these events. This neonate did not receive narcotics in the PACU. The patient remained in the PACU (12 hours) for continued apnea/bradycardia monitoring and was subsequently transported to the wards uneventfully. Continuous pulse oximetry was continued for an additional 24 hours. There were no further apneic episodes, and the patient’s remaining hospital course was uneventful. The neonate was discharged eight days after the procedure following completion of the anti-viral therapy. 

The second neonate was an ASA physical status 3E, preterm 37-week post-menstrual age female born via elective c-section on day seven of life who presented with a 24-hour history of bilious emesis. The neonate underwent an emergent exploratory laparotomy. The intraoperative course was uneventful. Approximately 73 minutes after arrival in the PACU, the patient’s hemoglobin oxygen saturation on pulse oximetry (SpO2) dropped to 72% with associated bradycardia to 35 beats per minute. Cardiopulmonary resuscitation was administered for three minutes. Following the return of spontaneous circulation, the neonate was upgraded to the Pediatric Intensive Care Unit (PICU) for further cardiopulmonary monitoring. Intubation or mechanical ventilation were not required. The neonate remained stable with no further apneic or bradycardic episodes. The neonate had one additional anaesthetic during the same hospital admission on day 10 of life (frenotomy and central venous catheter placement), tolerating this procedure well without any inotropic support. Post-operatively, she was transported to the PICU intubated and sedated due to concerns based on her previous apnea/bradycardia and arrest. Tracheal extubation was successful 12 hours after arrival in the PICU, and no other adverse events were observed during the rest of the hospital stay. Subsequently, the neonate was discharged 10 days after the initial surgery and presentation. Table 1 summarizes the recovery characteristics and outcomes of the two neonates who experienced postoperative apnea.

**Table 1:** Summary of recovery characteristics of neonates experienced apnea ETT=Endotracheal tube, PACU=Post-Anesthesia Care Unit, PICU- Pediatric Intensive Care Unit, ETT- Endotracheal Tube

	Case 1	Case 2
Age (days)	7	7
Gestational Age (weeks)	39	36
Post-Menstrual Age (weeks)	40	37
Sex	Male	Female
Surgery Duration (mins)	111	195
Surgical Procedure	Tunneled catheter placement	Exploratory laparotomy for mid gut malrotation with volvulus
ASA Physical Status	2	3E
Weight (kg)	3.48	2.04
Airway	ETT	ETT
Primary Anesthetic	General anesthesia	General anesthesia
Regional Anesthesia	None	None
Pre-medication	None	None
Intra-Operative Narcotic Use	None	Fentanyl – 5 mcg/kg
Neuromuscular Blocker	Succinylcholine – 1.7 mg/kg	Rocuronium – 1 mg/kg
Acetaminophen Use	None	None
Caffeine Use	None	None
Recovery in PACU	Yes	Yes
Time to Discharge from PACU	Stayed 12 hours in PACU for cardiorespiratory monitoring	73 minutes before upgrade to PICU
Opioids in PACU	None	None
Upgrade in Care Level	No	PICU
Description of Apnea Event	Several transient episodes (7) of 5-10 seconds - saturations improved following stimulation – saturations decreased to a nadir of 59% – returned to the floor	Apnea event occurred 2 hours post op – desaturation to 70s w/ bradycardia to 35 bpm - requiring cardiopulmonary resuscitation for 2 minutes no medications were given during resuscitation – subsequently upgraded to PICU – no further apneic episodes during hospitalization
Time to Discharge From Hospital Following Surgery (days)	8	10

Table 2 summarizes the risk factors identified in the neonates who experienced apnea/bradycardia compared to the rest of the study sample. The neonate with apnea had higher gestational age, PMA and chronological age but lower weight at the procedure and longer duration of the procedure. However, these did not achieve statistical significance, perhaps due to our low sample size. 

**Table 2:** Risk factors for post-operative apnea in neonates. Results are expressed and median IQR IQR=Inter Quartile Range

Characteristic	Group with no apneic events	Group with apneic events	Wilcoxon Rank Sum P-value
	Median (IQR)	Median (IQR)	
Gestational age at birth (weeks)	39.1 (37.6, 40.0)	37.5 (36.0, 39.0)	0.26
Post-menstrual age (weeks)	41.0 (38.6, 42.6)	38.5 (37.0, 40.0)	0.18
Weight (kg)	3.5 (2.9, 4.0)	2.8 (2.0, 3.5)	0.27
Age (days)	13.0 (3.0, 22.0)	7.0 (7.0, 7.0)	0.58
Duration of anesthesia	143.5 (110.0, 201.0)	153.0 (111.0, 195.0)	0.98

The results of the unadjusted bivariate analysis to identify predictors of recovery location are shown in Table 3, where ASA classification, preoperative anaemia, and known pathology were all found to be significant predictors (p<0.05). However, ASA classification was not included in the further multivariate analysis due to the low number of categories in this variable. 

**Table 3:** Results of Bivariate Analysis to Identify Predictors of Recovery Location post-operatively in neonates *Fisher’s exact test, PACU- Post-Anesthesia Care Unit, ASA- American Society of Anesthesiologist

Categorical			
Characteristic	Level	Post-op Recovery in PACU	P-value
ASA	1 1E 2 2E 3 4 5E	28 (93% 4 (67%) 48 (84%) 2 (100%) 23 (61%) 4 (57%) 1 (100%)	0.014*
Pre-operative anemia	No Unknown Yes	59 (69%) 47 (94%) 6 (86%)	0.002
Known pathology	No Yes	56 (95%) 56 (67%)	<0.001
Continuous			
Characteristic	Unadjusted odds ratio	95% confidence interval	P-value
Age (days)	1.17	1.09-1.25	<0.001
Duration of anesthesia (minutes)	0.99	0.98-1.00	0.003
Weight (kg)	1.85	1.04-3.29	0.035
Intra operative fentanyl (mcg per kg)	0.74	0.56-0.97	0.029
Amount of neuromuscular blocker (mg)	0.71	0.52-0.96	0.025
Time to discharge hospital (minutes)	0.96	0.93-0.99	0.027

Bivariate analysis determined that preoperative anaemia and co-morbidities (such as significant cardiac, respiratory, or neurologic co-morbidities) were significant predictors of recovery location. These factors were then combined in a multivariable logistic regression model. The amount of neuromuscular blocker given was not included in the further analysis due to low usage for the procedures included in this study. ASA classification was not included due to the low counts at some levels leading to model non-convergence. The significant factors for recovery location from the bivariate analysis were preoperative anaemia, known pathology, age, duration of anaesthesia, weight, intraoperative fentanyl, and amount of neuromuscular blocker. Reducing the model one factor at a time until all remaining predictors maintained p<0.05, both age and preoperative anaemia remained as significant predictors of recovery location, as shown in Table 4. 

**Table 4:** Results of Multivariable Analysis for post-operative Recovery Location in neonates

Characteristic	Comparison	Odds Ratio (95%CI)	P-value
Preoperative anemia (overall p=0.046)	Yes vs. No Unknown vs. Yes Unknown vs. No	3.4 (0.34-33.74) 1.4 (0.11-18.28) 4.8 (1.29-18.17)	0.020 0.79 0.30
Age (days)	Every 14 days	7.4 (2.80-19.31)	<0.001

The association with preoperative anaemia (overall p=0.046) appeared to be driven by comparing those with unknown preoperative anaemia vs no preoperative anaemia. The odds of recovery in the PACU were 3.4 times greater for those with preoperative anaemia than those without preoperative anaemia (OR=3.4, 95% CI=(0.34-33.74), p=0.02) when adjusted for age. Additionally, the odds of going to PACU for recovery were 7.4 times greater for every two weeks greater age (95% CI=(2.80-19.31), p<0.001), adjusted for preoperative anaemia. Our institutional policy (Appendix 1) is for children to remain under observation for a minimum of 12 hours following an apneic/bradycardic event. There was 100% compliance with our protocol. 

## Discussion

Post-operative apnea and bradycardia in infants remain a critical issue within the practice of pediatric anaesthesia [14]. Overall incidence described in the literature is variable, with preterm infant apnea rates of 10%-25% following general anaesthesia while term infant apnea rates are lower at approximately 4%-10% [5,8,17,18]. We report a similarly low overall incidence of 1.4% (2/144). For preterm infants, 0/20 (0%) experienced an apnea event, while for term infants, 2/124 (1.6%) had an event. Clinical characteristics of the two apnea cases were compared to the remaining patients, and although not statistically significant, these results are clinically meaningful and thus need to be evaluated in a larger sample with more events. In keeping with other literature, our study identified risk factors associated with post-operative apnea in infants, such as lower gestational age, lower post-menstrual age, and lower weight. We specifically focused on the neonatal population as this is the population at the highest risk for apnea among both preterm and full-term infant populations [8]. 

Our first presented case highlights that despite quantification of risk factors, there are cases that fall outside of predictive models. This neonate had no anaesthetic interventions such as opioid use (long or short-acting) or pre-medication that would have conferred an increased risk of post-operative apnea. Additionally, the neonate was a product of term pregnancy with appropriate weight for gestational age. Of note, the neonate was diagnosed with congenital herpes simplex infection. Although rare, infection with congenital herpes simplex virus (HSV) is a risk factor for apnea and may have been a factor in this case [19]. Patients with HSV exposure may develop various symptoms, including HSV encephalitis that often presents with apnea and lethargy. The neonate was undergoing a tunnelled line for treating an active HSV infection, and the absence of other traditional risk factors may suggest an alternative cause. This implies the need for continued vigilance in the recovery and the importance of adequate staff training. 

The second case presented had several additional apnea risk factors, including lower gestational/post-menstrual age, lower weight, and intraoperative narcotic use. Of note, this patient received neuromuscular blocking agents during induction but was not reversed at the discretion of the anaesthesia team after the case. In this instance, the neonate was noted to be active before extubation and had only received a single dose of rocuronium on induction for a procedure that was 195 minutes in length. The anaesthesia team deferred neuro-muscular blockade reversal based on clinical and neuromuscular monitoring (train-of-four) assessment. Although neuromuscular monitoring is unreliable in neonates, an inadequate reversal can contribute to apnea, and adequate reversal must be ensured in this age group [20,21].

The neonate met extubation criteria and remained stable for over one hour before the observed apneic episode. Following the episode, the neonate was upgraded to a higher level of care for continued cardio-respiratory monitoring. Experience from this apnea event guided future clinical decision making during the patient’s same hospitalization. While undergoing a subsequent anaesthetic, the patient remained intubated following the procedure without attempted extubation after the case. On follow-up 10 days later before charge, the neonate had no identifiable sequelae from the post-operative events. 

We have also examined the factors that impacted recovery location (PACU vs ICU) in this study. Statistical analysis suggested an association with preoperative anaemia (p=0.046) that appeared to be driven by the comparison of those with preoperative anaemia vs no preoperative anaemia (OR=3.4, 95% CI=(0.34-33.74), p=0.02). Interestingly, of those who did not have preoperative anaemia, more went to an ICU setting post-operatively than those with unknown haemoglobin or known anaemia. This suggests that anaemia is not an independent risk factor for transfer to NICU as post-operative location. Age also played a significant role, with the odds of going to PACU vs NICU/PICU for post-operative recovery were 7.4 times greater for every two weeks greater age (95% CI=(2.80-19.31), p<0.001). 

Although both neonates who experienced apnea cases in this study were term infants, prematurity with lower post-menstrual age is the strongest predictor of post-operative apnea in infants. Upon reaching the term, this risk decreases [17].

Although both patients presented in this study had positive outcomes, post-operative apnea or bradycardia events can have devastating consequences. Intraoperative caffeine has an established role in preventing post-operative apnea in infants and has been well studied in several prospective randomized control trials [11-13]. Additionally, caffeine is one of the safest and most cost-beneficial therapies in newborns regarding the treatment of apnea [12]. However, our incidence of apnea was now, questioning the need for routine intraoperative caffeine use. Of the 144 charts reviewed, only one patient received intraoperative caffeine. Accordingly, routine administration of intraoperative caffeine is not common at our institution. 

There are several limitations to this study. Our study is largely underpowered due to the low rate of identified apnea in the patient population at our institution. Perhaps, because of this, previously reported risk factors for neonatal apnea did not reach statistical significance. However, we aimed to evaluate the recovery characteristics in consecutive neonates undergoing general anaesthesia over two years. We chose to do this study following implementing a specific monitoring protocol in the neonatal age group. While apneas in premature infants are known, our study demonstrates that term neonates are also at risk of apnea.

Finally, since this is a retrospective chart review, there is a risk of missed documentation in our data based on self-reporting by the individual providers. However, any major events are unlikely to be missed. We have multiple capturing major adverse events data at our institute, including anaesthesia and nursing reports and a central adverse event reporting system.

## Conclusions

This study corroborates previous findings of predictive risk factors for post-anesthesia apnea in preterm and term neonates. Previously reported risk factors including low gestational/post-menstrual age, lower weight, and intraoperative narcotic use, were likely contributors to one of the apneic events in our study. A larger sample size is warranted to confirm a valid predictive model. Implementation of institutional protocols plays a major role in identifying, managing, and potentially preventing post-anesthesia apneas in the neonatal population. Standardized universal guidelines would help eliminate local variation in PACU monitoring and discharge criteria in this vulnerable age group.

## Appendices

Practice Advisory for Post-Anesthesia Monitoring in Ex-Premature and Term Infants Department of Anesthesiology and Perioperative Medicine 

All infants requiring anesthesia should preferentially be scheduled early in the day. 

Exceptions to these guidelines should be rare, and must be clearly justified. Consultation regarding such exceptions must include the attending anesthesiologist, attending surgeon, and the patient’s parent(s)/guardian(s).

Infants are monitored in the post-anesthesia recovery care areas according to our standard clinical criteria detailed in other policies, with the following enhancements:

Infant age

Minimum monitoring period

Instructions

Ex-premature < 60 weeks PMA

12 hours

see Procedure A

Term infants < 45 weeks PMA

12 hours

see Procedure A

Older infants, until 6 months of age

2 hours

see Procedure B

**Table 5:** Guidelines for post-anesthesia recovery care

Infant age	Minimum monitoring period	Instructions
Ex-premature < 60 weeks PMA	12 hours	see Procedure A
Term infants < 45 weeks PMA	12 hours	see Procedure A
Older infants, until 6 months of age	2 hours	see Procedure B

Procedure A:

Infants meeting the following criteria must be monitored longer than the standard PACU stay:

·      Premature (<37 weeks gestation) infants less than 60 post-menstrual weeks of age.

·      Full term (≥37 weeks gestation) infants less than 45 post-menstrual weeks of age.

·      Any infant on cardiorespiratory or apnea monitoring at home.

All infants meeting the above criteria must be monitored in the hospital as follows for a minimum of 12 hours post-anesthesia: 

a. Continuous pulse-oximetry 

b. Continuous apnea monitoring (e.g. ECG leads or other respiratory monitor).

c. Monitoring should be continuous, including throughout feeding. 

d. This 12-hour monitoring requirement clock restarts after any unexplained apneic event or bradycardic event. 

Based on current hospital processes, this monitoring will require ordering “continuous pulse oximetry” and “continuous ECG monitoring”. 

·      Inpatients: This monitoring can be done on all pediatric floors, Pediatric Intermediate Care Unit (PIMC), Neonatal Intensive Care Unit (NICU), and Pediatric Intensive Care Unit (PICU). 

·      Outpatients: Must be maintained as Outpatient Extended Recovery (OPER - length of stay <48 hours) status, or admitted to the hospital. Outpatients requiring monitoring cannot be Outpatient Admit (OPA - length of stay <24 hours). 

·      Depending on bed and staffing availability, these patients may be managed in the PACU, CH Pre-op, SDU, Radiology, pediatric floors, PIMC, or PICU. 

Procedure B:

Infants meeting the following criteria must be monitored longer than the standard PACU stay:

·      Any infant older than 45 or 60 weeks PCA, but still less than 6 months of age.

All infants meeting these criteria must be monitored in the hospital as follows for a minimum of 2 hours post-anesthesia: 

a. Continuous pulse-oximetry 

b. Monitoring should be continuous, including throughout feeding. 

c. If any apneic or bradycardic events are notes, then the infant should be monitored for a full 12 hours, as described above in Procedure A. 
